# Geographic variation in fungal diversity associated with leaf spot symptoms of *Coffea arabica* in Yunnan, China

**DOI:** 10.3389/fmicb.2025.1568029

**Published:** 2025-09-19

**Authors:** Xingfei Fu, Haohao Yu, Yaqi Li, Guiping Li, Xiaofei Bi, Yanan Li, Faguang Hu, Wenjiang Dong

**Affiliations:** ^1^Institute of Tropical and Subtropical Cash Crops, Yunnan Academy of Agricultural Sciences, Baoshan, China; ^2^Spice and Beverage Research Institute, Chinese Academy of Tropical Agricultural Sciences, Wanning, China; ^3^Yunnan Key Laboratory of Coffee, Baoshan, China

**Keywords:** *Coffea arabica*, fungal diversity, leaf spot, ITS sequencing, biocontrol

## Abstract

In China, the small grain coffee plants (*Coffea arabica*) are mainly cultivated in Yunnan province, yet the diversity of associated fungi remains poorly characterized. In this study we collected symptomatic leaves from 16 locations across Pu’er City and Xishuangbanna Dai Autonomous Prefecture (*n* = 48 samples, triplicate controls). Fungal communities were analyzed via ITS amplicon sequencing (Illumina MiSeq). We identified 3,638 fungal OTUs, dominated by Ascomycota (92%), including pathogens (*Colletotrichum gloeosporioides, Cercospora coniogrammes*), saprophytes, and beneficial entomopathogens (*Lecanicillium, Simplicillium*). The fungal communities showed significant geographical variation, with Pu’er City exhibiting a higher relative abundance of pathogenic fungi such as *Colletotrichum gloeosporioides* and *Cercospora coniogrammes*, while Xishuangbanna had a greater presence of beneficial entomopathogenic fungi such as *Lecanicillium* and *Simplicillium*. We classified abundant fungal OTUs into 48 different species colonizing leaves of coffee plants. Our core microbiome analysis revealed the presence of *Cercospora coniogrammes* (2%), the *Fusarium equiseti* of *Nectriaceae* family (5%), and the novel pathogenic fungi *Colletotrichum gloeosporioides* and *Cercospora coniogrammes.* Interestingly, we also identified the anti-phytopathogenic fungi belonging to the genus *Simplicillium* (9%) and entomopathogenic fungi known as *lecanicillium* (11%). This first report of *C. coniogrammes* and *C. gloeosporioides* in Yunnan coffee highlights the need for region-specific disease management. The prevalence of entomopathogenic fungi in Xishuangbanna suggests untapped biocontrol potential. Our data provide a foundation for monitoring leaf-associated fungi to improve crop resilience.

## 1 Introduction

The commercial production of coffee plants started in China during the 1980s. High market demand and more consumption of coffee have incentivized Chinese farmers to grow coffee plants instead of tea. Yunnan province has become a major coffee-producing region in China due to its fertile soil and, favorable environment (Chu R. et al., 2016: Neilson and Wang, 2019). Moreover, the growing awareness and demand for coffee among Chinese consumers have further driven the development of Yunnan’s coffee industry. It is estimated that Yunnan province alone produces 98% of China’s total coffee production, making it the largest coffee producing area in the country. Within Yunnan, Pu′er, Xishuang-banna, Baoshan, Dehong, Lincang and Wenshan are major coffee growing areas (Neilson and Wang, 2019). Although coffee has a significant impact on the earnings of farmers in developing countries, however, its productivity has been challenged due to persistent climatic and disease stress (Pham et al., 2019).

Coffee is susceptible to fungal infection at all stages of its growth, processing and storage, ultimately leading to a reduction in its quality [reviewed by Cui et al. (2020) and Asad et al. (2023)]. While Yunnan’s climate favors coffee cultivation, fungal diseases, particularly leaf spot pathogens, threaten productivity and bean quality. Leaf spot diseases, caused by fungi such as *Cercospora* and *Colletotrichum* species, lead to defoliation, yield loss, and secondary infections during post-harvest storage. Despite their economic impact, the diversity of fungi associated with coffee leaf spots in Yunnan remains poorly characterized, limiting targeted management strategies. Recent studies highlight the role of microbiome diversity in plant health, with endophytes and pathogens dynamically interacting on leaf surfaces. For example, entomopathogenic fungi like *Lecanicillium* can suppress foliar diseases, while pathogenic *Colletotrichum gloeosporioides* exacerbates leaf spot severity. However, most research in Yunnan has focused on soil or root microbiomes, neglecting the phyllosphere fungal community—a critical frontier for biocontrol discovery. Additional studies conducted in the Hainan province of China resulted in the identification of *Colletotrichum* species complex (Cao et al., 2019). Coffee anthracnose due to *Colletotrichum* species is increasing in Yunnan and Hainan provinces, leading to toxic effects in diseased coffee leaves and berries (Zheng et al., 2015). Brown leaf spot or cercospora leaf spot disease caused by *Cercospora coffeicola* in Brazil also affects the quality of coffee beverages and significantly reduces the coffee yield (Martins et al., 2008).

Based on their mode of infection or host interaction, the fungi can be divided into three broad categories, which include saprobes, pathogenic and endophytes (Santamaría and Bayman, 2005; Botrel et al., 2018; Da Alves Silva et al., 2020; Lu et al., 2022b). Saprophytes are considered neutral, as they do not cause disease in healthy plants. However, they cause the decaying of leaves after their detachment from the parent plants. Endophytes usually live inside the host in a synergistic mode without causing any damage or symptoms (Torres-Mendoza et al., 2020). Endophytic fungi are known to inhibit the growth of insects like coffee borers and can be potentially used to control pests (Vega et al., 2008; Goates and Mercier, 2009). They can complete their life cycle in their host. Endophytic fungi produce the nutrients that protect their hosts from stresses like high temperature and drought (Dubey et al., 2020). The experiments conducted by Pecundo et al. (2021) on Cycadales in China suggested that endophytic fungi showed antagonistic activities against the pathogenic fungi (Pecundo et al., 2021). The endophytic fungi showed a high inhibition potential (70–80%) of phytopathogenic fungi. Further experiments conducted on endophytic fungi demonstrated that they produce volatile organic compounds, which can kill the phytopathogenic fungi (Monteiro et al., 2017). In a recent study, the antimicrobial activity of volatile organic compounds of endophytic fungi isolated from *Coffea arabica* was observed (Monteiro et al., 2017). The volatile compounds produced by an endophytic fungus (*Muscodor coffeanum*) showed promising antifungal effects on *Aspergillus ochraceus*. Similarly, the fungicidal effect of endophytic fungi was also observed for *Fusarium verticilloides* inoculated on maize seeds. However, it is reported that endophytic fungi in coffee beans may transition to saprophytic or pathogenic (saprobes) after harvesting due to environmental changes (Huang et al., 2021). Examples of such species are known from *Fusarium*, *Penicillium* and *Aspergillus*, which produce toxins in agricultural products (Miller and Trenholm, 1994). These toxins reduce the quality of coffee beans and are reportedly neurotoxic or carcinogenic in nature (Proctor et al., 2018).

The literature suggests that microbial associations vary across different production systems. For example, the experiments on root microbiomes of coffee plants suggested that microbial communities vary across different management practices and environmental conditions. A pyrosequencing-based study of bacteria on a coffee farm in Brazil suggested that organic matter in the soil, the type of plantation and changes in plantation sites have a greater impact on the community structure of nitrogen-fixing bacteria (Caldwell et al., 2015). The leaf spectrum characteristics like photosynthetic capacity, nutrient contents and life span play a crucial role in plant’s interaction with endophytic microbes. The inter- and intra-micro-environmental interaction of plants influences the microbial communities inhabiting the aerial and root parts. Similarly, Yu et al. (2025) showed that inoculating the *Morchella importuna* mycosphere with *Pseudomonas chlororaphis* increased the α-diversity of the soil fungal community and suppressed the abundance of the pathogen *Paecilomyces penicillatus*, leading to reduced white mold disease incidence and increased morel yield. This suggests that fungal communities play critical roles in disease suppression not only in green plants but also in mushroom crops and that manipulation of beneficial microorganisms could be a broadly applicable strategy in the biological control of disease (Yu et al., 2025).

Fungi-specific marker genes, commonly called an internal transcribed spacer (ITS) region of the rDNA genes have revealed a high degree of genetic diversity in the fungal pathogens. These ITS sequences are used for fungal identification and operational taxonomy unit (OTU) delineation. High-throughput sequencing allows amplification and sequencing of ITS clusters from complex environmental samples. Different studies have used next-generation sequencing and OTU analysis to explore the genetic diversity and population dynamics of the leaf rust disease of coffee (Vieira et al., 2019; Talhinhas et al., 2017). Some investigations have also explored the potential of endophytic fungi as biocontrol agents against fungal diseases by utilizing OTU analysis (Bongiorno et al., 2016). This study was designed to understand the variation in the fungal communities of coffee leaves growing at two different locations in Yunnan province of China. Our findings provide a foundation for region-specific disease management and highlight candidate biocontrol agents. This work also underscores the need to monitor leaf-associated fungi as indicators of crop health in Yunnan’s rapidly expanding coffee industry.

## 2 Materials and methods

### 2.1 Collection of leaf samples, DNA isolation and PCR

In January 2024, infected leaves of *Coffea arabica* (CIFC 7963 variety) showing leaf spot symptoms and visible fungal mycelia were collected from 16 different locations in Yunnan Province, China, covering both Pu’er City and Xishuangbanna Dai Autonomous Prefecture—two ecologically distinct regions with known differences in altitude (Pu’er: ~1,300 m; Xishuangbanna: ~550 m), microclimate, and agricultural practices (Neilson and Wang, 2019) (Supplementary Table S1). At each site, triplicate samples were collected (total *n* = 48). To minimize contamination, sampling was conducted using sterile gloves and medical-grade stainless steel scissors, which were sterilized between samples using 70% ethanol and flame. Approximately 5 g of infected leaf tissue was excised and immediately placed in sterile 2 mL screw-cap tubes stored in ice bags during transport to the laboratory. In the laboratory, the leaf samples underwent a surface sterilization protocol: 95% ethanol (60 s), 3% sodium hypochlorite (3 min), followed by another rinse in 95% ethanol (30 s), and a final wash with sterile distilled water. After air drying, samples were placed on potato dextrose agar (PDA) plates as described previously (Tibpromma et al., 2018; Lu et al., 2022a). For fungal DNA extraction, hyphal mats were scraped from PDA cultures. The PDA plates were kept at 28 °C and were observed daily for mycelium growth. The fungal colonies were separated and transferred to new tubes for DNA isolation. For DNA extraction, the samples were ground to a fine powder in the presence of liquid nitrogen at an intermediate speed in a homogenizer (PowerLyzer 24 Homogenizer, Qiagen, Germantown, MD, US). Total DNA from these samples was extracted using the DNAeasy Plant Kit (Qiagen, Germantown, MD, USA). The purified DNA was eluted in 100 μL elution buffer and quantified by Nanodrop (2000, Thermo Fisher Scientific, MA, USA). The quality of extracted DNA was tested by running it on 1.2% agarose gel. To monitor contamination, a negative extraction control (blank tube) was included in each batch. For amplification of ribosomal RNA, the internal transcribed spacer (ITS) region was used as a target sequence for the ITS1 and 4 primers (Rehner and Buckley, 2005). Briefly, high-fidelity *Pfu* DNA polymerase was used for the amplification of the target region (Quanshijin). In a 25 μL PCR reaction, 100 ng DNA was used as a template and 25 cycles were run in the PCR machine as described earlier (Tibpromma et al., 2018). Primers with 10 picomoles concentrations were used with 50 °C annealing temperature. Each run included a no-template control (NTC) to detect reagent contamination. No amplification was observed in negative controls. The PCR products were further purified by magnetic beads (Vazyme VAHTSTM DNA Clean Beads). The re-covered PCR products were quantified through the fluorescence quantification method (Bustin, 2005) using the Microplate reader (Quant-iT PicoGreen dsDNA Assay Kit; Microplate reader-BioTek, FLx800). In order to control for the potential contamination, we included extraction blanks and negative controls in PCR reactions (no-template controls) in every batch of DNA extraction and amplification. No visible amplification was observed in any of the negative controls. All biological replicates (triplicate samples per site) were sequenced and analyzed independently to preserve intra-site variation and to confirm reproducibility of fungal community profiles. Each of the three biological replicates collected per site was processed, sequenced, and analyzed independently, rather than pooled, to capture intra-site variability. This approach allowed us to evaluate the reproducibility of fungal community profiles across replicates within the same location.

### 2.2 ITS library generation and sequence analysis

The ITS sequencing libraries were prepared using Illumina’s TruSeq Nano DNA LT Library Prep Kit. In the first step end re-pairing of amplified products was conducted to remove any overhangs at the 5′ end (End Repair Mix 2). The adenine nucleotide (A base) was added to prevent self-ligation of the PCR products. The adapter sequences were added at the 5′ end followed by PCR amplification for enrichment purposes (Illumina’s TruSeq Nano DNA LT Library Prep Kit). The enriched PCR products were purified before the final sequencing reaction (BECKMAN AM-Pure XP Beads). Before the sequencing reaction, the quality of PCR products was checked on the Agilent Bioanalyzer (Agilent High Sensitivity DNA Kit; Agilent Technologies, USA). The libraries having more than 2 nM PCR DNA concentrations were sequenced through the Illumina MiSeq platform using PE300 settings.

### 2.3 Quality control and Bioinformatic analysis

The original sequencing reactions were checked for quality and subjected to trimming. Subsequently, the sequences underwent denoising and chimera removal through the DADA2 plugin (Callahan et al., 2016). The ITS reads of fungi were processed by following the DAD2 ITS Pipeline Workflow 1.8.^1^ For the ITS sequences, UNITE was selected by the default Database (Release 8.0, https://unite.ut.ee/) (Kõljalg et al., 2013). For each set of amplicon sequence variant (ASV) or OTU database, we used QIIME2’s classify-sklearn algorithm^2^ (Bokulich et al., 2018). To assign the features for each ASV sequence or representative sequences of each OTU, we used species annotation using the pre-trained Naive Bayes classifier in QIIME2 software using default parameters. We used the BLASTn database first and compared the results with the fungi-specific BROCC algorithm (Nilsson et al., 2006)^3^ to obtain annotation information based on the default parameters.

### 2.4 Statistical analysis and data presentation

The R software (R Foundation for Statistical Computing R Core Team, 2014) was used for statistical analysis, including generating the taxa identification table (Supplementary Table S2). It was also utilized to create histograms illustrating the identification results at each classification level, allowing for a visual comparison of OTU numbers across different samples.

We used ggtree package of R language to draw a microbial taxonomic hierarchical tree and added the grouped abundance data of each taxa node to the graph in the form of a pie chart (Yu et al., 2017). Due to multiple taxa in samples, we set the relative abundance threshold of 0.1% to display at the taxonomical level. For visual observation and species comparisons, we used the Krona Species Composition Diagram (https://github.com/marbl/Krona/wiki: V2.7 Ondov et al., 2011). Subsequently, we examined alpha diversity (Evenness and richness) based on OTU clusters by employing Chao1 Shannon and Simpson indices (Hughes et al., 2001). We also explored the beta diversity (differences in species composition) by calculating the Bray-Curtis distance (Bray and Curtis, 1957). The results of beta diversity analysis were further confirmed by principal coordinate analysis (PCoA) (Ramette, 2007).

### 2.5 Species differential analysis

After exploring the differences in microbial community composition, we further analyzed the differential distribution of OTUs in the samples by employing the plotrix package (Lemon, 2006). To further explore the taxonomic distribution of species in each area of the petal diagram, a histogram was developed corresponding to the number of OTUs at the phylum and genus levels. We compared the differences in species abundance composition between the samples by drawing a heat map (Kolde and Kolde, 2015). The fungal OTUs were assigned to ecological functional groups (pathogens, saprotrophs, endophytes, and entomopathogens) based on published literature and curated resources. Taxa reported as plant pathogens in previous studies (e.g., *Colletotrichum gloeosporioides*, *Cercospora coniogrammes*) were classified as pathogenic (Chen et al., 2005; Guatimosim et al., 2016). Saprotrophic taxa (e.g., *Periconia*, *Montagnula*) were annotated following Yang et al. (2022) and Du et al. (2021). Entomopathogenic and endophytic fungi such as *Lecanicillium* and *Simplicillium* were categorized according to Vega et al. (2008) and Setiawati et al. (2021). Where ambiguity existed, functional assignments were made conservatively and annotated as “unclassified.” This literature-based approach ensured that ecological functions were assigned only when supported by prior peer-reviewed reports.

For identification of the core fungal microbiome, we defined “core taxa” as OTUs present in ≥70% of all samples with a minimum relative abundance of 0.1%. This prevalence-based threshold has been commonly applied in microbial ecology studies to capture consistently occurring taxa while excluding rare or spurious sequences. The prevalence and abundance of core taxa across sites were summarized in Supplementary Table S3.

### 2.6 Metagenome sequence analysis

For phylum and class level differences in the samples, we used OTUs data, which were significantly different at the genus and species levels. We used the unflattened OTU values in pairwise comparison to determine the significance of the difference. Additionally, we used the linear discriminant analysis effect size (Segata et al., 2011). The LEfSe was run through the Galaxy online analysis platform^4^ to perform differential analysis on all taxonomic levels simultaneously.

### 2.7 Association network analysis

We assessed the co-occurrence of fungal communities driven by changes in sampling location (Faust and Raes, 2012). For this analysis, samples with less than 5 OTUs were filtered out. Briefly, OTU nodes in the taxon between two points represented the association networks of fungal species. The SparCC algorithm was used to construct a correlation matrix as described earlier (Friedman and Alm, 2012). Based on the abundance of OTU nodes, the top 50 nodes with average abundance values were extracted to construct a dominant species sub-network.

### 2.8 Metabolic pathway statistics

From the same samples, metabolic pathways for primary and secondary metabolites were analyzed using the MetaCyc metabolic pathway database (Caspi et al., 2020). In the first step, the abundance of metabolic pathways was calculated. After obtaining the abundance data on metabolites, we tried to find their significant differences among the groups (Charlson et al., 2011). Finally, based on significant differences in metabolites among the groups, the stratified sample metabolic pathway abundance table was used to analyze the species composition of the differential pathways (Franzosa et al., 2018).

## 3 Results

### 3.1 Quality and characteristics of sequence datasets

**Figure 1:**
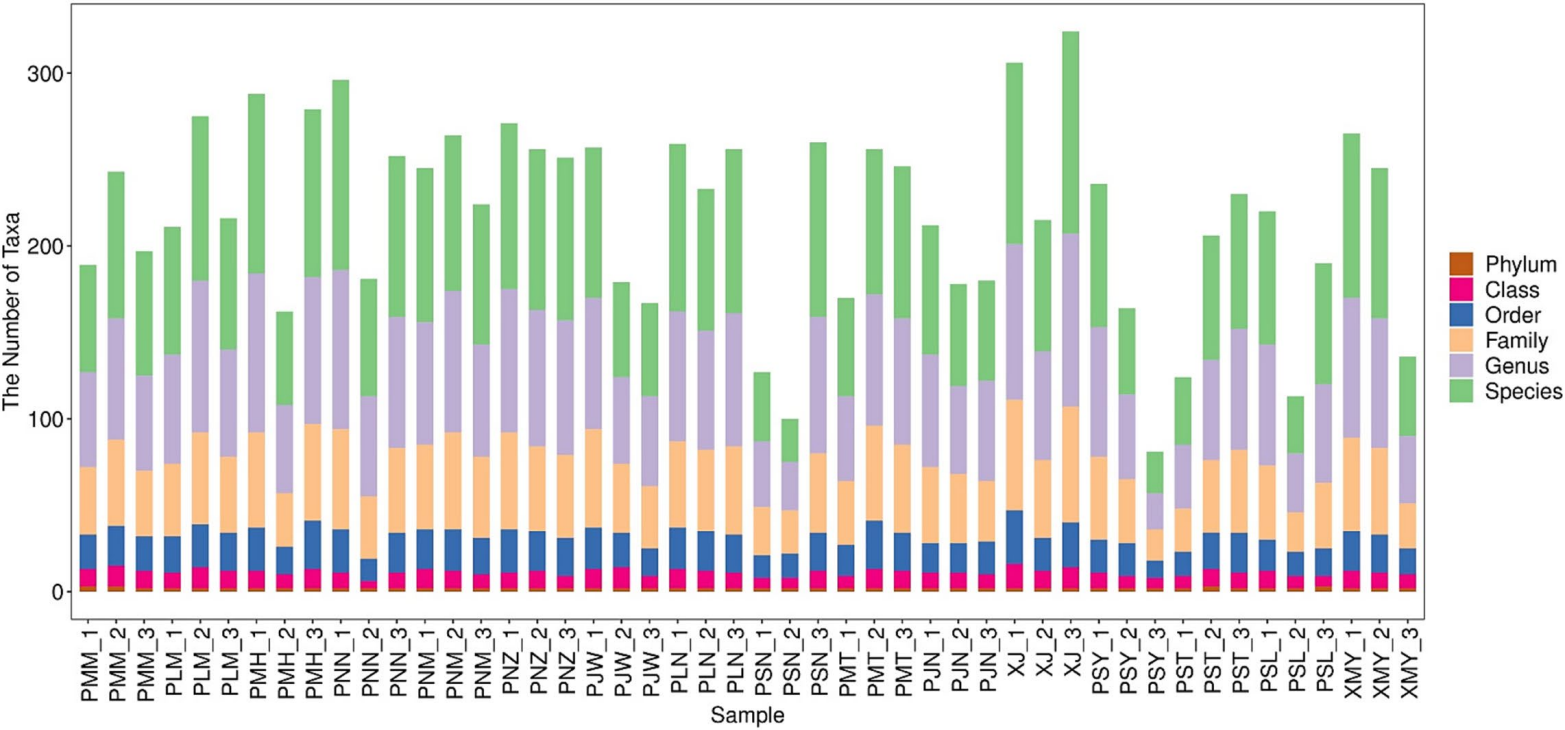
Taxonomic composition of fungal communities in coffee leaf samples across Yunnan, China. The *X*-axis represents the samples from different locations; while *Y*-axis represents the count of taxa at Phylum, class, order, family, genus and species level.

A total of 39,581 full-length ITS sequences were filtered out after denoising, splicing and removing the chimeras. The average length of the ITS region amplified in this study was 299 bp with a range of 228–439 bp (Supplementary Figure S1). Each library was separately analyzed for denoising. The entire short read sequences and singletons (taxa represented once only) were removed and subsequently, the sequencing volume of each sample was counted and presented in Supplementary Table S3. To assign the features to each OTU, the BLASTn results were represented along with the nucleotide database of the BROCC algorithm. To show the sequence annotation results, species identification results were statistically analyzed. It is worth noting that due to the wide variety of microorganisms, some of the microbes could not be identified at the species level; therefore, they were mentioned as unclassified. After the species annotation, an average number of 111 species belonging to ~24 genera were identified (Supplementary Table S3). In the species dataset, we were able to identify a minimum of 26 species and a maximum of 182 species (Figure 1).

### 3.2 Community composition of fungi isolated from coffee plants

**Figure 2:**
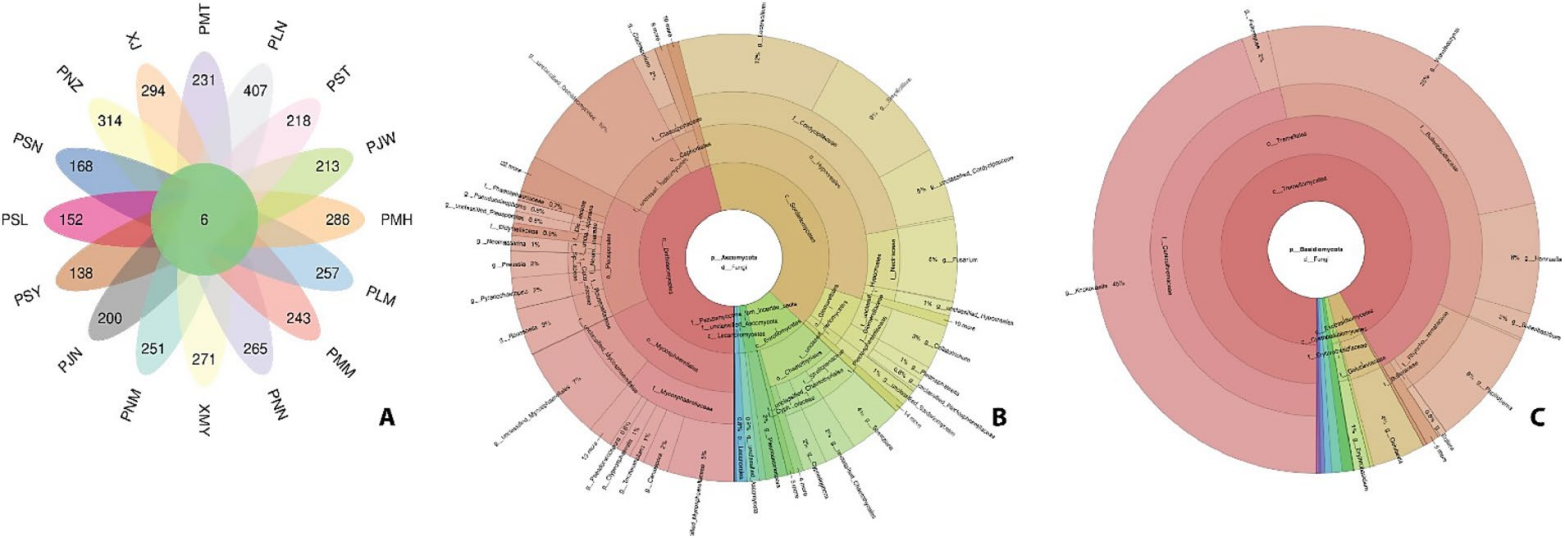
Fungal communit structure across Yunnan coffee leaf samples. **(A)** Petal diagram of OTUs in the leaf samples. Each ellipse represents a sample group. The overlapping region in the middle represents the total OTUs of all groups. **(B)** Relative abundance of Ascomycota isolated from *Coffea arabica* leaves. **(C)**. Relative abundance of Basidiomycota.

The OTU data suggested the presence of a variable number of species in each sample (Figure 2A). We wanted to check the relative abundance of different fungi at each taxonomic level. Based on the cumulative data from all the samples, Ascomycota were the most abundant detected phyla (92% of total sequences), followed by Basidiomycota (4%) and unidentified phyla (4%). The most frequently detected class of Ascomycota was Dothidiomycetes, which accounted for 46% of all the sequences. This class was dominated by families, *Mycosphaerellaceae* (43% unclassified, 16% cercospora, 10% teratoramularia, 9% Clypeosphaerella, 6% Pseudocer-cospora, 4% Acrodontium, 4% Xenoramularia, 3% Neoceratosperma) *Roussoellaceae*, *Cucurbitariaceae*, Spo*r*omeaceae, *Neomassarinaceae*, *Didymellaceae*, *Dictyosporia*-ceae, *Phaeosphaeriaceae*, *Cladosporiaceae*, *Capnodiaceae* (81% unclassified, 15% Leptoxyphium and 4% Capnodium), *Cordycipitaceae* (45% Lecanicillium, 35% Simplicillium, and 19% unclassified), *Nectriaceae* (99% Fusarium), *Glomerellaceae* (100% Collecto-trichum), *Plectosphaerellaceae*, *Strelitzianaceae* and *Cyphellophoraceae* (Figure 2B; Supplementary Figure S2).

Relative abundance data of sequences revealed that Basidiomycota was the second most frequently detected phyla of total sequences (Figure 2C). It was mainly dominated by the class Tremellomycetes (92% of Basidiomycota). It included the species members from the family, *Cuniculitremaceae*, *Bulleribasidiaceae, Rhynchogastremataceae, Bulleraceae, Trimor-phomycetaceae, Golubeviaceae, Erythrobasidiaceae* and some unclassified Basidiomycota (0.7%).

### 3.3 Phylogeny of species

**Figure 3:**
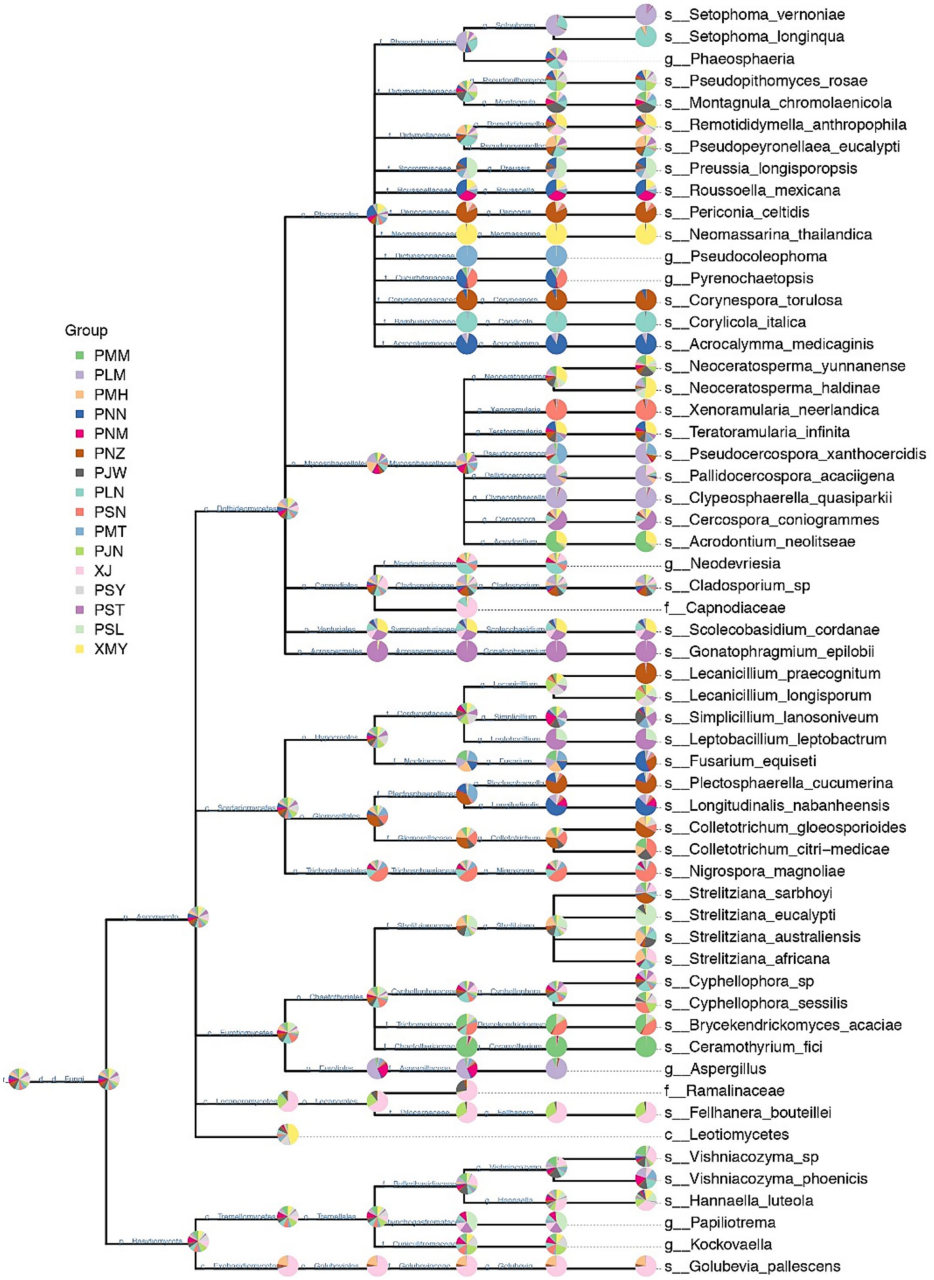
Hierarchical taxonomic distribution of fungal communities in Yunnan coffee leaves. The pie chart (threshold 0.1%) of each branch node of the classification level trees shows the proportion of taxon in each sample.

After the above-mentioned genus-level classification, we were successfully able to identify 48 abundant species. However, in the relative species abundance data, the species-level identification could not be assigned to 10 OTUs. The phylogenetic tree at the species level was constructed at a microbial taxonomic hierarchy and grouped abundance data of each taxa node to the graph in the form of a pie chart (Figure 3). Since there are so many taxa in some samples, we set the relative abundance thresholds of 0.1% and only displayed taxa nodes whose relative abundance is greater than the thresholds at the same taxonomic level. For Ascomycota, we confirmed 44 OTUs at the species level, while for Basidiomycota only 4 OTUs were identified at the species level. In the phylogram of Ascomycota, most of the OTUs in each genus were confirmed at the species level. However, in certain cases, the classification at the species level was not possible. For example, in the order Pleosporales, 13 out of 16 OTUs were successfully confirmed at species level. In the family *Mycospaerellaceae*, all the OTUs (9 out of 9) were supported at the species-level identification. A single species in each of the families *Cladosporiaceae*, Sympoventuriaceae and Acrospermaceae was confirmed.

### 3.4 Diversity in microbial/fungal communities

**Figure 4:**
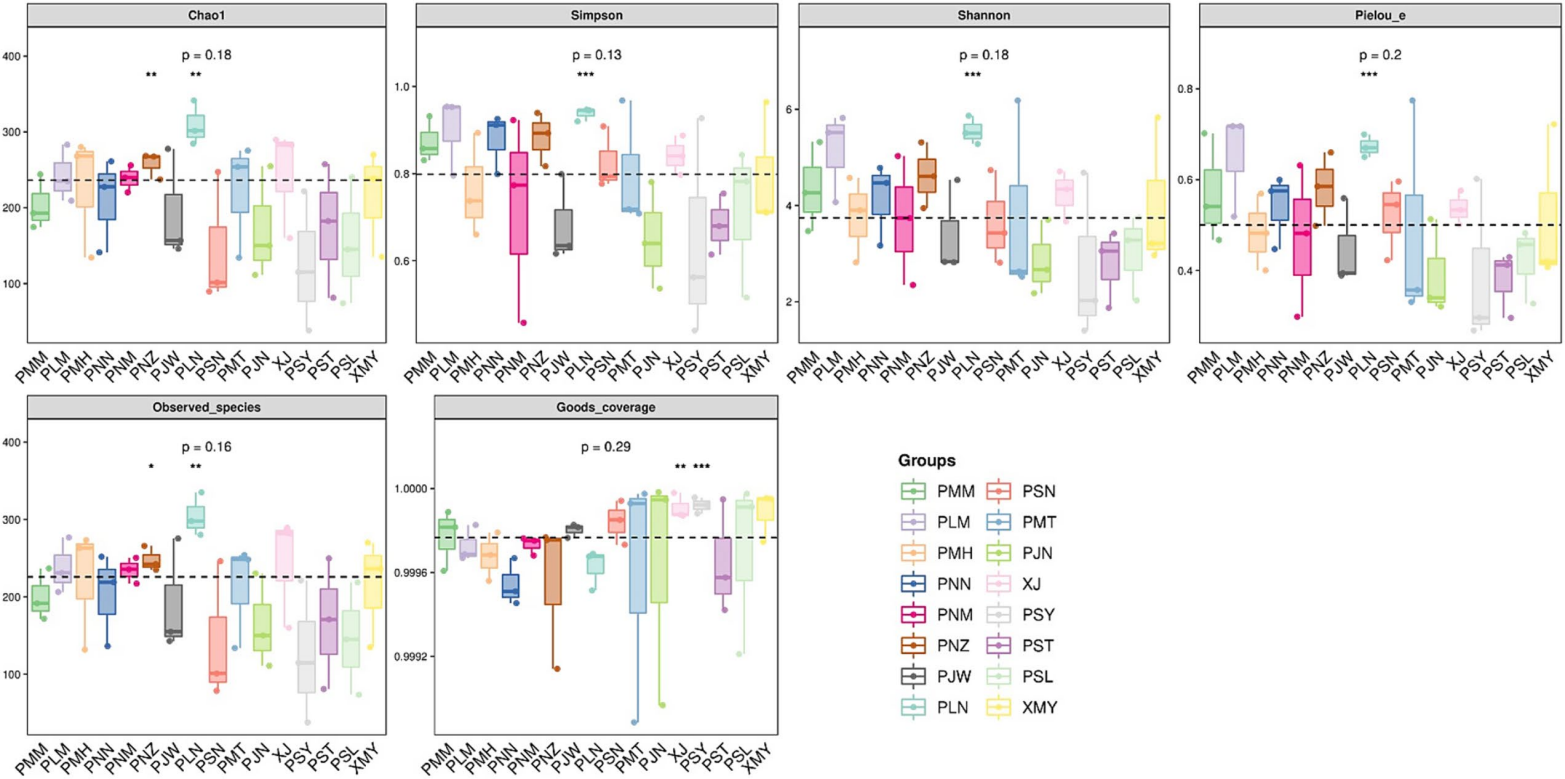
Regional differences in fungal alpha diversity indices: Each panel corresponds to an alpha diversity index, which is marked in the gray area at the top. In each panel, the abscissa is the group label, and the ordinate is the value of the corresponding alpha diversity index.

**Figure 5:**
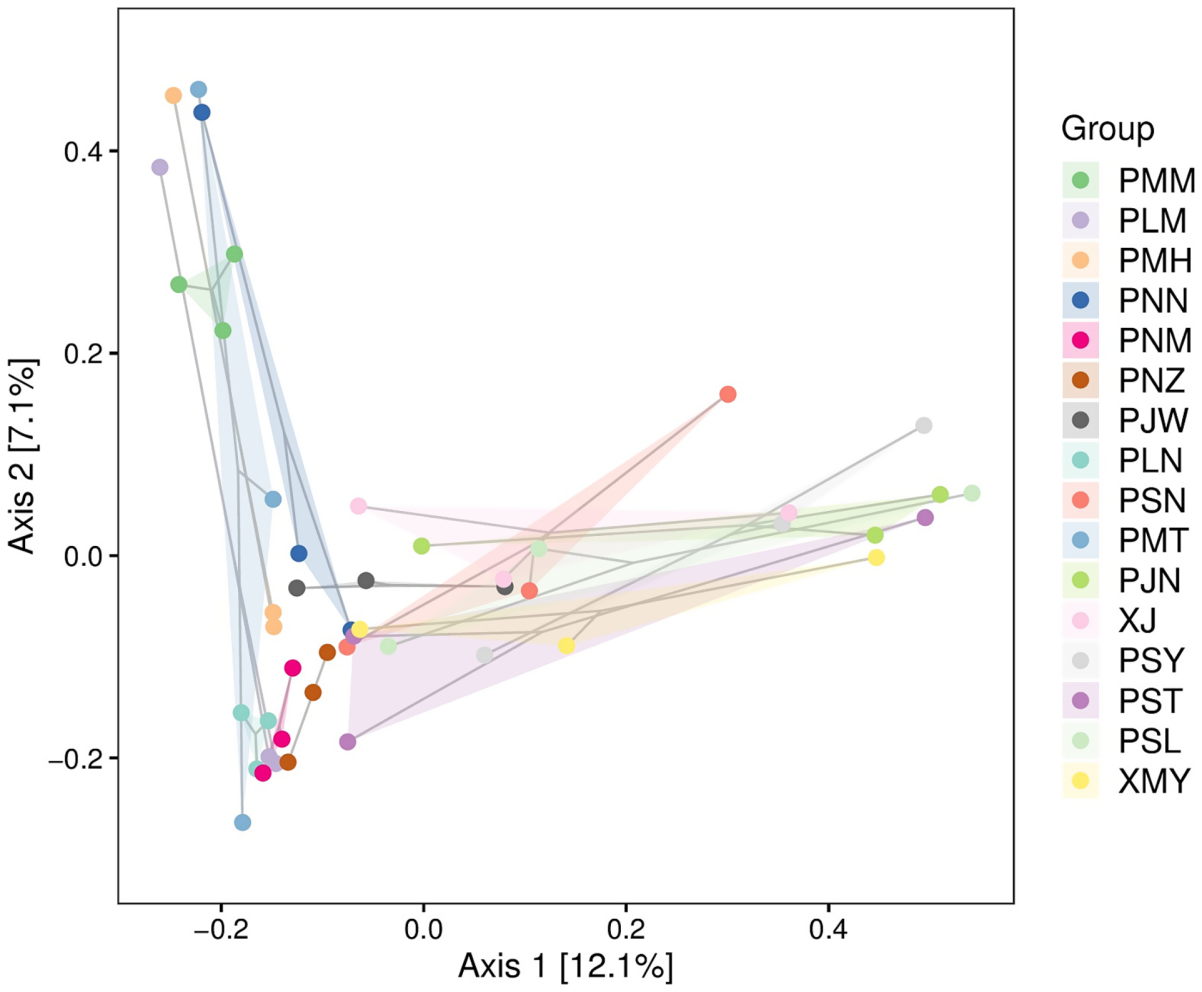
PCA of fungal communities in coffee leaves from Pu’er (P-codes) and Xishuangbanna (X-codes), based on Bray–Curtis dissimilarity. The Axis 1 and Axis 2 represent the greater variation among the samples. Each sample is represented with a unique color. The proximity of the points to each other suggests the degree of similarity in species composition, while those that farther apart are more dissimilar.

To represent the species richness, evenness and diversity indices we used the Alpha diversity index (Figure 4). The box plots for Chao1 alpha diversity indices indicated a range of values across different sample groups with minor levels of differences (*p*-value = 0.18). Similarly, the box plot values of Simpson (species belonging to the same group, *p*-value = 0.13), Shannon (abundance and evenness, *p*-value = 0.18) and Pielou (species richness and evenness, *p*-value = 0.2) indicated variability across the groups with some outliers. In addition to alpha diversity indices, we used principal coordinate analysis (PCoA) to understand the species composition differences across the changing localities. The beta diversity analysis indicated that, by changing the location of coffee sampling sites in Yunnan the degree of variability in each species group is increased (Figure 5). In Figure 5, Principal Component Analysis (PCA) revealed significant geographical structuring of fungal communities across the two coffee-growing regions. Axis 1, explaining 12.1% of the total variance, primarily separates the sampling locations by city: Pu′er City clusters on one side, while Xishuangbanna clusters on the opposite side. This dominant axis reflects the key ecological divergence between regions, with the Pu′er cluster characterized by a higher relative abundance of plant pathogenic fungi (*Colletotrichum gloeosporioides* and *Cercospora coniogrammes*), and the Xishuangbanna cluster associated with a higher relative abundance of beneficial entomopathogenic fungi (*Lecanicillium*, *Simplicillium*). Axis 2 explains a smaller proportion of the variance (7.1%) and likely captures subregional variation within each city, potentially driven by localized environmental conditions or management practices. The spread of locations along Axis 2 (values ranging from approximately −0.2 to 0.4) indicates moderate heterogeneity in fungal composition among sites within each major region. Collectively, the PCA plot clearly demonstrates the significant geographical differentiation in coffee-associated fungal communities between Pu′er City and Xishuangbanna, as identified in our analysis.

### 3.5 Association network analysis

**Figure 6:**
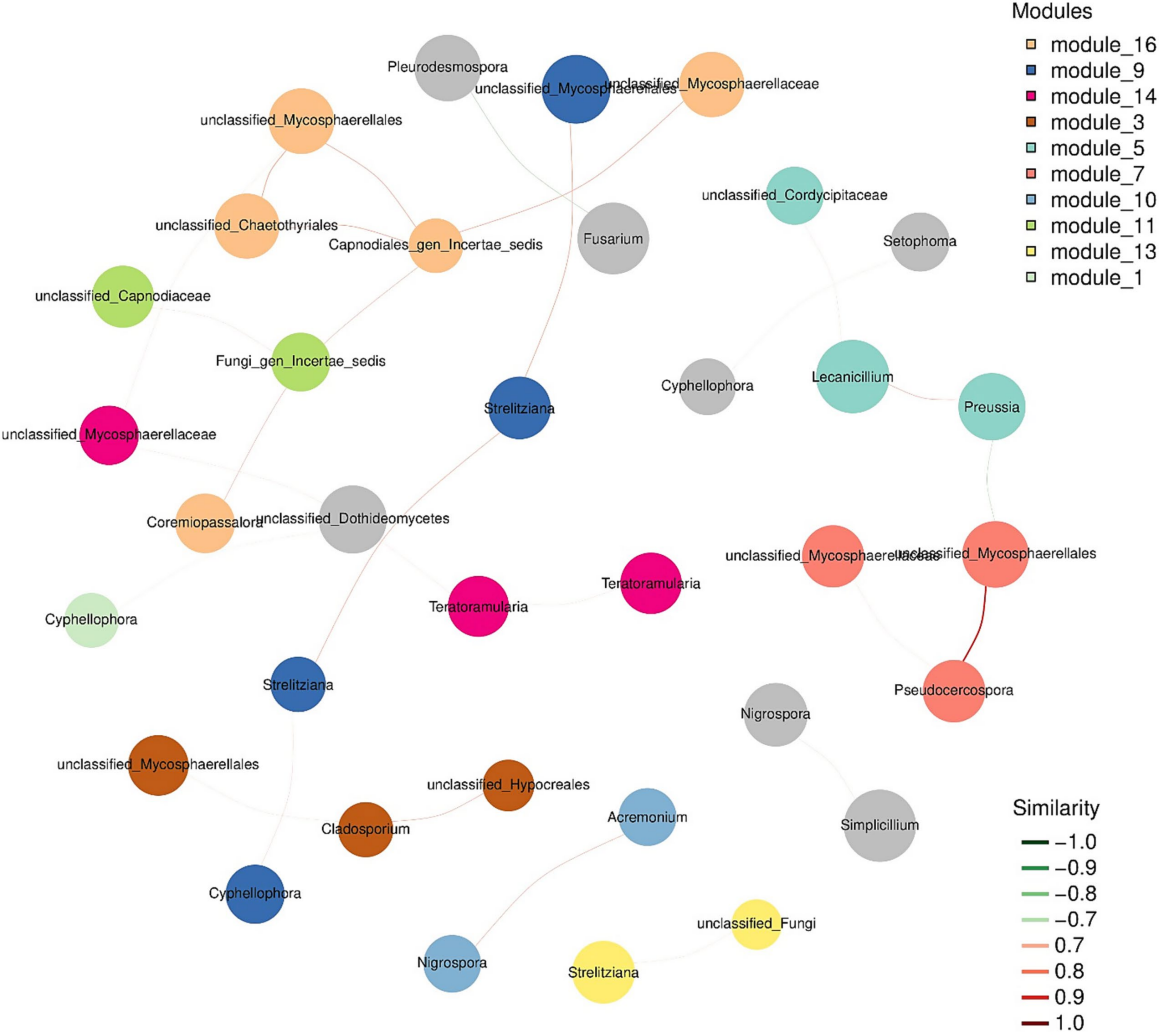
Modular dominant species seed network diagram. Nodes represent the OTU in the sample. The size of the node is proportional to its abundance [in log2(CPM/n)]. The figure only shows the top 50 ASV/OTUs with the average abundance of the sample. Different colors identify the modules with the most nodes in the top 10 (or less); the edge between nodes indicates that there is a correlation between the two connected nodes, with the red line indicating positive correlation and the green line indicating negative correlation.

Subsequently, we wanted to assess the co-occurrence of microbial diversity with each sampling location. For this purpose, we constructed the association network analysis from the top 50 most abundant OTUs from the sequence data. However, in the network association analysis, the species representation changed due to variable sampling location. Therefore, we wanted to address the presence of the most dominant species in all the samples through an association network. The co-occurrence of species at sub-network level is presented in Figure 6. The network analysis suggested that entomopathogenic fungi belonging to Cordycipitaceae are the most dominant species in different samples. It is clear from the network association that both pathogenic and endophytic fungi (Table 1) are present and can be correlated with each other at relatively weaker levels, with some exceptions of pathogenic fungi. For example, the presence of pathogenic fungi *Pseudocercospora xanthocercidis* is highly correlated with other members of *Mycosphaerellacea* family (Figure 6). A relatively weaker correlation was observed between member species of *Strelitzianaceae* and *Mycosphaerellacea*. In the case of entomopathogenic fungi like *Lecanicillium* and *Simplicillium*, we could not identify any level of correlation for their abundance levels. Similarly, members of pathogenic species like *Colletotrichum*, *Cercospora*, and *Fusarium* did not show any significant correlation with each other.

**Table 1:** Presence of different species in the leaf samples of coffee plants.

Family	Species name	Mode of nutrition
Phaeosphaeriaceae	*Setophoma vernoniae*	Pathogenic
Phaeosphaeriaceae	*Setophoma_longinqua*	Pathogenic
Didymosphaeriaceae	*Pseudopithomyces rosae*	Pathogenic
Didymosphaeriaceae	*Montagnula chromolaenicola*	Saprophytic
Didymellaceae	*Remotididymella anthropophila*	Pathogenic
Didymellaceae	*Pseudopeyronellaea eucalypti*	Pathogenic
Sporomiaceae	*Preussia longisporopsis*	Found on goat dung
Roussoellaceae	*Roussoella mexicana*	Pathogen
Periconiaceae	*Periconia celtidis*	Saprophytic
Neomassarinaceae	*Neomassarina thailandica*	Saprophytic
Corynesporaceae	*Corynespora torulosa*	Pathogenic
Bambusicolaceae	*Corylicola italica*	Saprophytic
Acrocalymmaceae	*Acrocalymma medicaginis*	Pathogenic
Mycosphaerellacea	*Neoceratosperma yunnanense*	Pathogenic
Mycosphaerellacea	*Neoceratosperma haldinae*	Pathogenic
Mycosphaerellacea	*Xenoramularia neerlandica*	Unclassified
Mycosphaerellacea	*Teratoramularia infinita*	Unclassified
Mycosphaerellacea	*Pseudocercospora xanthocercidis*	Pathogenic
Mycosphaerellacea	*Pallidocercospora acaciigena*	Pathogenic
Mycosphaerellacea	*Clypeosphaerella quasiparkii*	Unclassified
Mycosphaerellacea	*Cercospora coniogrammes*	Pathogenic
Mycosphaerellacea	*Acrodontium neolitseae*	Unclassified
Sympoventuriaceae	*Scolecobasidium cordanae*	Pathogenic
Acrospermaceae	*Gonatophragmium epilobii*	Unclassified
Cordycipitaceae	*Lecanicillium praecognitum*	Entomopathogenic
Cordycipitaceae	*Lecanicillium longisporum*	Entomopathogenic
Cordycipitaceae	*Simplicillium lanosoniveum*	Endophytic/entomopathogenic
Cordycipitaceae	*Leptobacillium leptobactrum*	Unclassified
Nectriaceae	*Fusarium equiseti*	Pathogenic
Plectosphaerellaceae	*Plectosphaerella cucumerina*	Pathogenic
Plectosphaerellaceae	*Longitudinalis nabanheensis*	Unclassified
Glomerellaceae	*Colletotrichum gloeosporioides*	Pathogenic
Glomerellaceae	*Colletotrichum citri−medicae*	Unclassified
Trichosphaeriaceae	*Nigrospora magnoliae*	Pathogenic
Strelitzianaceae	*Strelitziana sarbhoyi*	Unclassified
Strelitzianaceae	*Strelitziana eucalypti*	Unclassified
Strelitzianaceae	*Strelitziana australiensis*	Unclassified
Strelitzianaceae	*Strelitziana africana*	Unclassified
Cyphellophoraceae	*Cyphellophora sessilis*	Saprophytic
Trichomeriaceae	*Brycekendrickomyces acaciae*	Unclassified
Chaetothyriaceae	*Ceramothyrium fici*	Pathogenic
Pilocarpaceae	*Fellhanera bouteillei*	Unclassified
Bulleribasidiaceae	*Vishniacozyma phoenicis*	Yeast, symbiont (Drosophila gut)
Bulleribasidiaceae	*Hannaella luteola*	Yeast, saprophytic
Golubeviaceae	*Golubevia pallescens*	Yeast, saprophytic

### 3.6 Prediction of functional potential reveals differential metabolite accumulation

**Figure 7:**
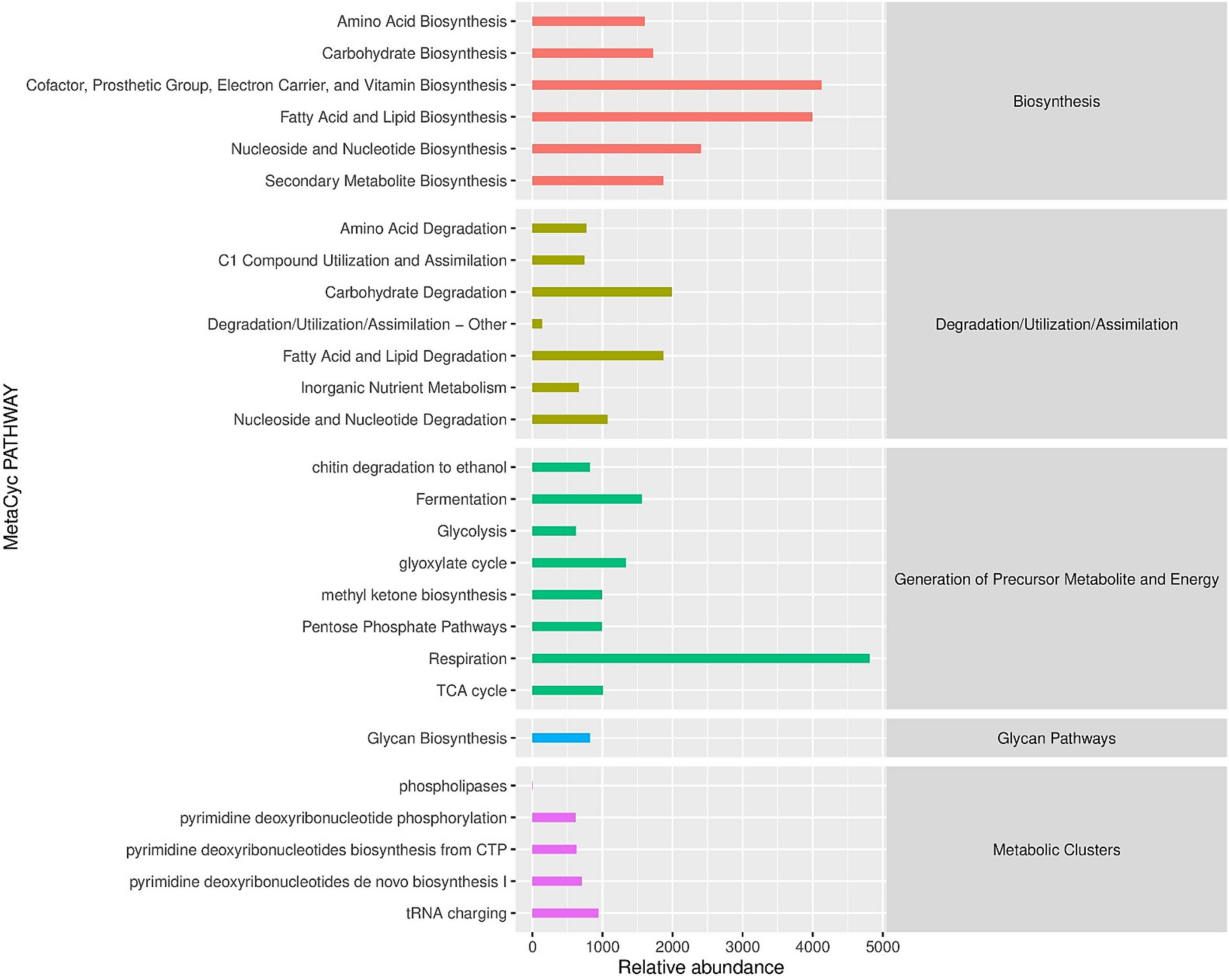
Region-stratified functional profiles of coffee-associated fungi. The abscissa is the abundance of the functional pathway (unit is per million PWY), the ordinate is the functional pathway at the second classification level of MetaCyc, and the far right is the first-level pathway to which the pathway belongs. Shown here is the average abundance across all samples.

At the biochemical level, we assessed the potentially important metabolites in the leaf samples. We used MetaCyc, for the estimation of primary and secondary metabolites (Figure 7). The MetaCyc analysis showed the relative abundance of pathways categorized into three main sections: Biosynthesis, Degradation and Generation of precursor metabolites and energy. Amino acids and lipid biosynthesis showed relatively increased abundance, while their degradation level was relatively low. In contrast, carbohydrate biosynthesis indicated a relatively low abundance compared to amino acid biosynthesis. However, carbohydrate degradation showed moderate abundance. Similarly, nucleosides and nucleotides biosynthesis and their degradation exhibited a lower abundance than lipid biosynthesis but higher than carbohydrates. In addition to functional pathway analysis, we used LEfSe (Linear Discriminant Analysis Effect Size) to identify fungal taxa that were differentially abundant between the two regions. The analysis revealed that the pathogenic genera *Colletotrichum* and *Cercospora* were significantly enriched in Pu’er City samples (LDA score >3.5, *p* < 0.05), whereas the entomopathogenic genera *Lecanicillium* and *Simplicillium* were significantly enriched in Xishuangbanna (LDA score >3.2, *p* < 0.05). These taxa represent potential indicator species, with *Colletotrichum* and *Cercospora* serving as markers of pathogen-dominated communities, and *Lecanicillium* and *Simplicillium* as potential suppressors or beneficial taxa associated with disease moderation.

## 4 Discussion

In China, Yunnan’s coffee production has become a driving factor for the transition from a less significant commercial crop to a global specialty in coffee. Its huge consumption at domestic and global levels indicates coffee’s growing impact on the Chinese economy (Zhang et al., 2013). The coffee farmers in the tropics face several problems in yield and quality due to climate change, ever-increasing pests and fungal diseases (Harvey et al., 2014; Veloso et al., 2023; Pham et al., 2019). Fungal diseases of the coffee plant, like leaf spot can destroy the coffee crop in hot and humid areas like Yunnan. Fungal endophytes in coffee plants can potentially improve the uptake of important nutrients like phosphorus and nitrogen. Endophytes from coffee plants are also reported to cope with drought and salinity conditions (Redman et al., 2002).

In this study, we found several pathogenic fungi including *Cercospora coniogrammes*, which is a novel fungi and previously reported from ferns in Korea (Guatimosim et al., 2016). We also discovered *Colletotrichum gloeosporioides*, which is a weak pathogen of ripped coffee berries (Chen et al., 2005). Nowadays, exploration of the microbiome associated with coffee beans and soil allows us to understand the pathogenic or beneficial microbes, which can be used for the improvement of beverage quality in coffee (Brioschi Junior et al., 2021). In this analysis of ITS region datasets, we reported the pattern of mycobiome in leaves of coffee plants growing in Pu′er City and Xishuangbanna Dai areas of Yunnan province. Previous experiments suggest that coffee varieties and their associated microorganisms have stronger adaptation in certain regions than others due to climatic factors (Veloso et al., 2020). The composition of fungal communities varied notably between the two locations. Ascomycota was the most dominant phylum in both regions, but at different proportions, with Dothideomycetes being the most frequently detected class. Specific fungal species such as *Cercospora coniogrammes* and *Colletotrichum gloeosporioides*, both known coffee pathogens, were more prevalent in Pu’er City, indicating that environmental stressors may favor their proliferation. In contrast, entomopathogenic fungi such as *Lecanicillium* and *Simplicillium* were more abundant in Xishuangbanna Dai, suggesting that microclimatic conditions in this area might support a more complex ecological interaction between fungi and their hosts. Our study revealed distinct patterns in fungal diversity and composition between the coffee-growing regions of Pu’er City and Xishuangbanna Dai, underscoring the role of phylogeographic factors in shaping microbial communities. Pu’er City, characterized by relatively cooler temperatures and higher altitudes, exhibited a higher fungal diversity index compared to Xishuangbanna Dai. The increased fungal diversity in Pu’er may be attributed to more stable temperature variations and reduced humidity, which promote a greater coexistence of both beneficial and pathogenic fungal species. In contrast, Xishuangbanna Dai, with its tropical climate, higher humidity, and warmer temperatures, showed a dominance of pathogenic fungi such as *Colletotrichum gloeosporioides* and *Cercospora coniogrammes*. These species are known to thrive in warm and humid environments, correlating with the higher disease prevalence reported in this region.

This is a novel study to evaluate the influence of altitude factors on the mycobiome inhabiting coffee leaves. Previously, the microbiota inhabiting coffee fruits and soil and the fermentation process of coffee were explored (Zhao et al., 2018; Júnior et al., 2019; Oliveira et al., 2013; Lee et al., 2015). In this study, we found significant differences in the diversity and structure of fungi in leaf samples, indicating that changes in microclimatic conditions can alter the coffee microbiota (Veloso et al., 2020). Indeed, it is already established that variation and interaction in topographic factors, like temperature change, presence of nitrogen and carbon contents, and intensity of light, can influence coffee production (Chu H. et al., 2016; Karungi et al., 2018). Although the ITS region is commonly used for fungal barcoding, it has certain limitations that require careful interpretation. While it effectively distinguishes species, measuring variation within species can be challenging.

Based on the OTU richness and evenness results, the Ascomycota was the most abundant phylum, comprising 92% of the sequence data. Basidiomycota was the second most abundant phylum (4%) at a relative abundance level. Our results are in agreement with previous studies, where a higher relative abundance of Ascomycota was observed as compared to Basidiomycota on soil fungal communities of Colombian coffee plantations (Ochoa-Henriquez et al., 2024). Interestingly, within Ascomycota, Dothideomycetes was the most frequently detected class, accounting for 46% of all sequences. The abundance of Dothideomycetes on coffee leaves reflects their significance with both beneficial and pathogenic members. Our results are consistent with the previous findings that within Dothideomycetes the genera like *Cercospora*, *Colletotrichum*, *Pseudocercospora* and *Fusarium* are the major coffee pathogens that can strongly impact the quality and production of coffee beverages (Guerra-Guimarães et al., 2023; De Sousa et al., 2021). Detection of *Lecanicillium* and *Simplicillium* species belonging to class Sordariomycetes is noteworthy due to their potential as entomopathogenic and mycoparasitic fungi. These genera are known for complex ecological interactions with other fungi and insects, which may contribute to natural biocontrol mechanisms in the coffee leaf environment (Wang et al., 2020; Setiawati et al., 2021). As a matter of fact, Basidiomycota and Ascomycota are comprised of a large number of species with a wide range of adaptations and lifestyles (Wang et al., 2021). Although fungi belonging to Mortierellomycota are also reported along with ubiquitous Ascomycota and Basidiomycota, however, in this study, we did not find a similar pattern. The network analysis for the co-occurrence of fungal species highlighted that entomopathogenic fungi belonging to the Cordycipitaceae family are the most dominant species. The entomopathogenic fungi exhibited a stronger correlation with each other compared to the pathogenic fungi.

Saprophytes were detected with low prevalence and abundance at each sample collection site. Among the saprophytes we identified, *Periconia celtidis, Montagnula chromolaenicola, Corylicola italica, Neomassarina thailandica, Hannaella luteola* and *Golubevia pallescens* (Table 1). Several saprophytic fungi like Periconia and Montagnula have a wide distribution and are reported from dead leaves and twigs of different hosts, including coffee plants (Yang et al., 2022; Du et al., 2021). The literature suggests that saprophytes play an important role in the recycling of nutrients and the decomposition of debris or dead plant material.

The metabolic and pathway analysis suggested a preferential accumulation of lipids and amino acids over carbohydrates in the coffee leaves. These metabolites act as a precursor to secondary metabolites and contribute to the quality of coffee extracts. The previous studies suggested that the accumulation of phenolic and volatile compounds and alkaloids can contribute to high-quality aroma (Rodrigues et al., 2021; Castro-Moretti et al., 2023).

## 5 Conclusion

This study provides key insights into the coffee leaf mycobiome in Yunnan province of China and its relationship with pathogens, potential bio-control agents and environmental factors. The study found significant differences in the structure and diversity of fungi, indicating the impact of microclimate. It is suggested that future research should focus on the inoculation of endophytes on the seedlings to improve the early stages of plant development. The information generated through mycobiome can be helpful in disease management and quality improvement strategies.

## Data Availability

The sequencing data are available at NCBI SRA: PRJNA1144603. All other data are available within the manuscript or upon reasonable request to the corresponding authors.

## Footnotes

^1^ https://benjjneb.github.io/dada2/ITS_workflow.Html

^2^ https://github.com/QIIME2/q2-feature-classifier

^3^ https://github.com/kylebittinger/q2-brocc#the-brocc-algorithm

^4^ http://huttenhower.sph.harvard.edu/galaxy/

## Supplementary material

SUPPLEMENTARY TABLE S1 Table representing the sample collection sites. The leaf samples were collected from 16 different locations. The latitude coordinates for each location are provided. Each location was sampled in triplicates.

SUPPLEMENTARY TABLE S2 The table describes the total number of filtered Amplicon sequence variants (ASVs). The genus level taxonomy is assigned to each ASV with high confidence value.

SUPPLEMENTARY TABLE S3 Taxonomic distribution of different taxa at each taxonomic level. Number of species in each sample are presented in the last column.

SUPPLEMENTARY FIGURE S1 Length distribution of amplified ITS region sequences.

SUPPLEMENTARY FIGURE S2 Abundance of different fungal taxa.
