# Supplementary material for: Geographic variation in fungal diversity associated with leaf spot symptoms of Coffea arabica in Yunnan, China
Source: Front Microbiol. 2025 Sep 19;16:1568029. doi: 10.3389/fmicb.2025.1568029 (PMC12491272; doi:10.3389/fmicb.2025.1568029)
Supplement: SUPPLEMENTARY TABLE S1 — Table representing the sample collection sites. The leaf samples were collected from 16 different locations. The latitude coordinates for each location are provided. Each location was sampled in triplicates. [file Table_1.DOCX]

**Table S1:** Table representing the sample collection sites. The leaf samples were collected from 16 different locations. The latitude coordinates for each location are provided. Each location was sampled in triplicates.

| **NO.** | **Collection area** | **Longitude and latitude, altitude** | **Sampling codes** | **Replications of samples** |
| --- | --- | --- | --- | --- |
| 1 | Pu'er City Menglian Dai and Lahu Autonomous County Mengma Town | E99°29′27.64″；N22°17′08.80″；海拔1331±5m | PMM | PMM-1 |
| 2 |  |  | PMM | PMM-2 |
| 3 |  |  | PMM | PMM-3 |
| 4 | Pu'er City The Nanling Mountain Township, Lancang County | E100°07′32.99″；N22°39′39.19″；海拔1004±5m | PLM | PLM-1 |
| 5 |  |  | PLM | PLM-2 |
| 6 |  |  | PLM | PLM-3 |
| 7 | Pu'er City Menglian Dai and Lahu Autonomous County Hong'an Lianda Coffee Estate | E99°30′00.06″；N22°22′39.19″；海拔1493±5m | PMH | PMH-1 |
| 8 |  |  | PMH | PMH-2 |
| 9 |  |  | PMH | PMH-3 |
| 10 | Pu'er City Ning'er County Ning'er Town | E100°57′33.33″；N23°11′31.79″；海拔945±5m | PNN | PNN-1 |
| 11 |  |  | PNN | PNN-2 |
| 12 |  |  | PNN | PNN-3 |
| 13 | Pu'er City Ning'er County Mohei Town | E101°19′56.73″；N23°14′23.29″；海拔886±5m | PNM | PNM-1 |
| 14 |  |  | PNM | PNM-2 |
| 15 |  |  | PNM | PNM-3 |
| 16 | Pu'er City Ning'er County Dehua Town | E100°51′57.28″；N23°09′34.87″；海拔1308±5m | PNZ | PNZ-1 |
| 17 |  |  | PNZ | PNZ-2 |
| 18 |  |  | PNZ | PNZ-3 |
| 19 | Pu'er City Weiyuan Town, Jinggu County | E100°49′40.44″；N23°56′56.14″；海拔822±5m | PJW | PJW-1 |
| 20 |  |  | PJW | PJW-2 |
| 21 |  |  | PJW | PJW-3 |
| 22 | Pu'er City Nuozhadu Town, Lancang County | E100°23′20.75″；N22°32′26.29″；海拔964±5m | PLN | PLN-1 |
| 23 |  |  | PLN | PLN-2 |
| 24 |  |  | PLN | PLN-3 |
| 25 | Pu'er City Simao District Nanping Town | E100°59′32.16″；N22°37′31.79″；海拔1023±5m | PSN | PSN-1 |
| 26 |  |  | PSN | PSN-2 |
| 27 |  |  | PSN | PSN-3 |
| 28 | Pu'er City Tongguan Town, Mojiang County | E101°20′13.97″；N23°14′33.30″；海拔865±5m | PMT | PMT-1 |
| 29 |  |  | PMT | PMT-2 |
| 30 |  |  | PMT | PMT-3 |
| 31 | Pu'er CityZhengdong Town, Jiangcheng County | E101°29′05.16″；N22°33′36.80″；海拔1065±5m | PJN | PJN-1 |
| 32 |  |  | PJN | PJN-2 |
| 33 |  |  | PJN | PJN-3 |
| 34 | Dai Autonomous Prefecture of Xishuangbanna Puwen Town, Jinghong City | E101°01′15.83″；N22°32′54.95″；海拔1155±5m | XJ | XJ-1 |
| 35 |  |  | XJ | XJ-2 |
| 36 |  |  | XJ | XJ-3 |
| 37 | Pu'er City Simao District LYixiang Town | E101°23′43.28″；N22°39′38.51″；海拔913±5m | PSY | PSY-1 |
| 38 |  |  | PSY | PSY-2 |
| 39 |  |  | PSY | PSY-3 |
| 40 | Pu'er City Simao District Longtan Township | E100°40′15.00″；N22°41′23.57″；海拔1066±5m | PST | PST-1 |
| 41 |  |  | PST | PST-2 |
| 42 |  |  | PST | PST-3 |
| 43 | Pu'er City Simao District Liushun Town | E100°41′25.69″；N22°34′16.10″；海拔992±5m | PSL | PSL-1 |
| 44 |  |  | PSL | PSL-2 |
| 45 |  |  | PSL | PSL-3 |
| 46 | Dai Autonomous Prefecture of Xishuangbanna Yiwu Town, Mengla County | E101°32′19.93″；N22°20′16.70″；海拔1007±5m | XMY | XMY-1 |
| 47 |  |  | XMY | XMY-2 |
| 48 |  |  | XMY | XMY-3 |
