# Supplementary figures and images for: Geographic variation in fungal diversity associated with leaf spot symptoms of Coffea arabica in Yunnan, China
Source: Front Microbiol. 2025 Sep 19;16:1568029. doi: 10.3389/fmicb.2025.1568029 (PMC12491272; doi:10.3389/fmicb.2025.1568029)

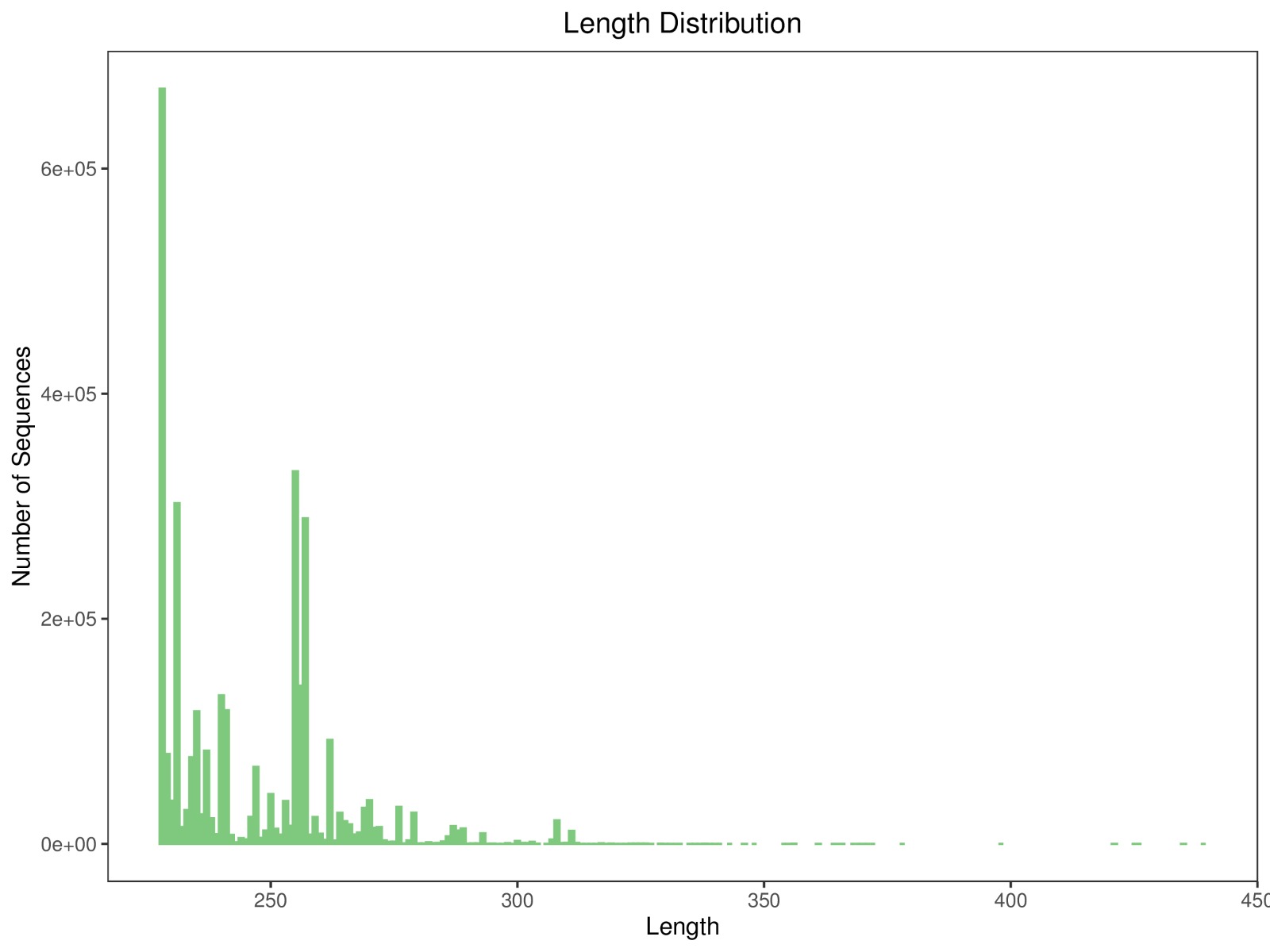

Supplement: SUPPLEMENTARY FIGURE S1 — Length distribution of amplified ITS region sequences. [file Image_1.JPEG]

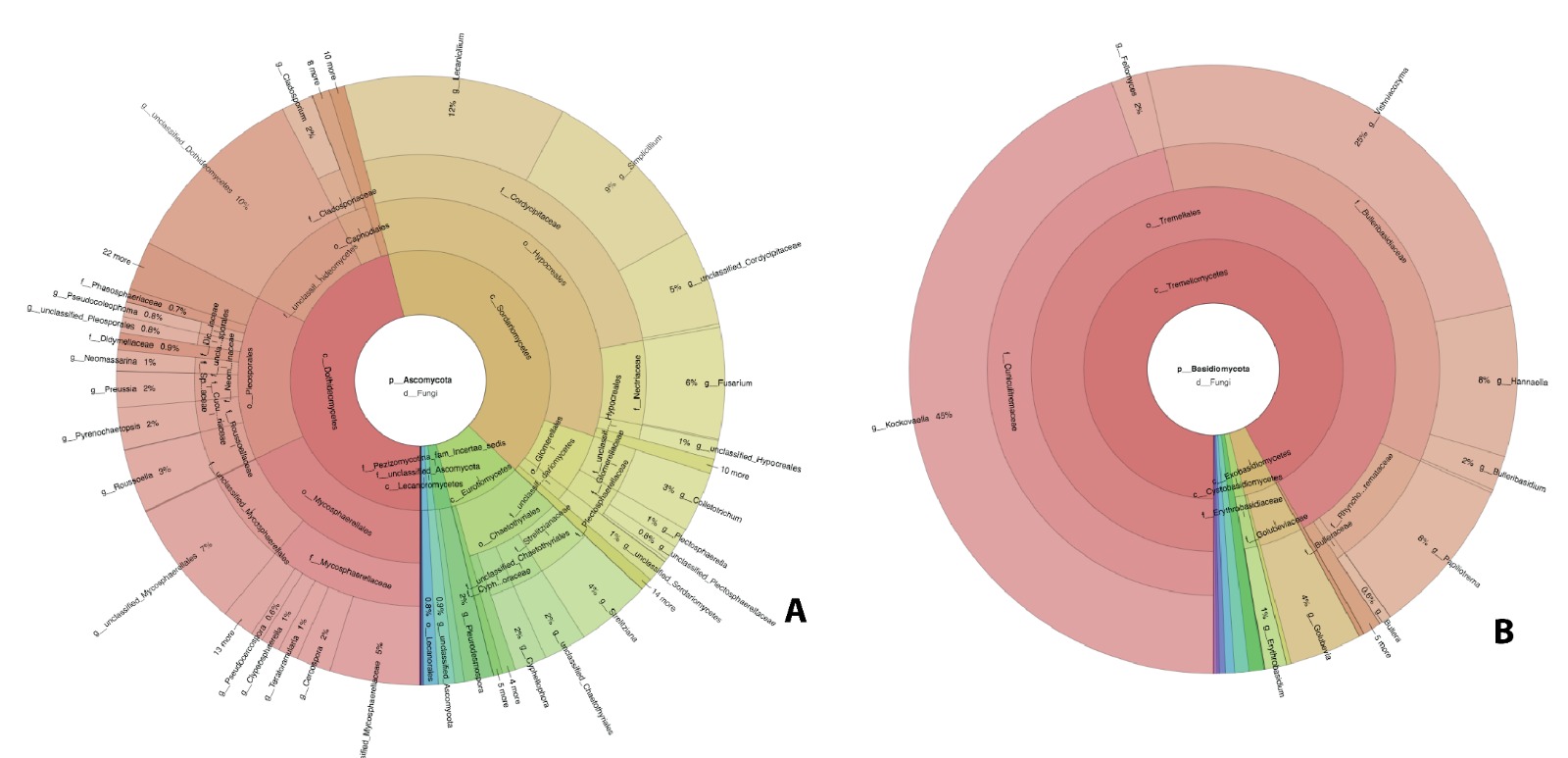

Supplement: SUPPLEMENTARY FIGURE S2 — Abundance of different fungal taxa. [file Image_2.JPEG]
